# A New Galactoglucomannan from the Mycelium of the Medicinal Parasitic Fungus *Cordyceps cicadae* and Its Immunomodulatory Activity In Vitro and In Vivo

**DOI:** 10.3390/molecules28093867

**Published:** 2023-05-04

**Authors:** Fei Gao, Lingling Luo, Leifang Zhang

**Affiliations:** College of Food and Pharmacy, Zhejiang Ocean University, 1 South Haida Road, Zhoushan 316022, China

**Keywords:** *Cordyceps cicadae*, polysaccharide, immunomodulatory activity, macrophages, cell membrane receptor

## Abstract

A new galactoglucomannan (C-0-1) was purified from the medicinal parasitic fungus of *Cordyceps cicadae* using an anion-exchange column and gel permeation column. The results of high-performance liquid chromatography and high-performance gel permeation chromatography indicated that C-0-1 consists of galactose, glucose, and mannose in a ratio of 5:1:4 and has a molecular weight of 23.3 kDa. The combined structural elucidation analysis methods including partial acid hydrolysis, methylation analysis, and NMR experiments revealed that C-0-1 was a comb-like polysaccharide with a core structure including (1→2)-α-D-Man*p* residues in the backbone and branches at *O*-6 of the main chain. (1→4)-α-D-Glc*p*, (1→2)-β-D-Gal*f*, (1→2,6)-β-D-Gal*f*, and terminal β-Gal*f* were located at the side chains. An in vitro experiment using RAW 264.7 cells indicated that C-0-1 exhibits good immunomodulatory activity by enhancing inducible nitric oxide synthase secretion and the production of some major inflammatory cytokines. On inhibiting the cytokine production using anti-pattern recognition receptors antibodies, it was revealed that the activation of macrophages is mainly carried out by C-0-1 through the mannose receptor. Toll-like receptor 4 and Toll-like receptor 2 were also involved in this identification process. An in vivo experiment on immunosuppressive mice treated with cyclophosphamide indicated that C-0-1 improves the secretion of serum-related cytokines (IFN-γ, TNF-α, IL-2, IL-4, and IL-10) and affects the balance of T helper cells Th1/Th2. Given the structural and bioactivity similarity between *Cordyceps cicadae* and *Cordyceps sinensis*, we can conclude that *Cordyceps cicadae* could be used as an important medicinal fungus like *Cordyceps sinensis*.

## 1. Introduction

*Cordyceps* (family Clavicipitaceae, Ascomycotina) is an intriguing fungal genus that is known for its characteristic parasitic nature, and is famous for its use as food, tonic, and medicine [1,2]. *Cordyceps sinensis*, as the most popular species in the *Cordyceps* genus, is well studied, explored, and has been widely used as a traditional Chinese medicine for over 300 years [3,4]. In Asia, *C. sinensis* has been regarded as a precious medicinal material with various pharmacological effects. It could be used to treat diverse chronic diseases, have immunomodulatory effect and anti-cancer activity, and is beneficial to hepatic and renal functions [5,6,7]. The growth of *C. sinensis* requires a seriously restricted habitat in the Tibetan plateau. Further, a combination of declining yields and a strong demand leads to the degradation of this natural resource and high prices year by year [8]. Several methods such as artificial cultivation and fermentation have been researched to meet the use demands for *C. sinensis* [9]. However, despite the technological advances, the artificial cultivation of *C. sinensis* has proven to be difficult because of its strict cultivation requirements. Thus, *C. sinensis* is still a rare and precious commodity. A new commodity with similar benefits and uses is needed.

*Cordyceps cicadae* is also an entomopathogenic fungus that belongs to the genus *Cordyceps*. It grows inside the nymph of hosts, such as *Cicada flammata* Distant and *Platylomia pieli Kato*, and forms fruiting bodies on the surfaces of these insects [10]. It has been used as a food and in traditional Chinese medicine to treat malaria, palpitations, cancer, diabetes, and chronic kidney diseases for centuries [11]. Although *C. cicadae* is one of the oldest traditional Chinese medicine with abundant resources in South China, it has not gained as much attention as *C. sinensis*. Research on the components of *C. cicadae* over the past two decades has revealed that it has similar bioactive compounds to *C. sinensis*, including nucleosides, sterols, cyclic dipeptides, polysaccharides, and aromatic compounds [12,13]. These studies on the chemical profile and bioactivity suggest that *C. cicadae* can be used as an alternative resource to *C. sinensis* [14,15,16]. Polysaccharides are one of the important biologically active components of *C. sinensis* and have attracted more and more attention from scientific communities. In this study, we researched the structure and immunomodulatory activity in vitro and in vivo of the polysaccharides in *C. cicadae*, compared the similarities and differences in terms of structure and function with reported polysaccharides from *C. sinensis*, and analyzed the possibility of using medicinal fungus *C. cicadae* as a candidate of *C. sinensis* based on the polysaccharides.

## 2. Results and Discussion

### 2.1. Extraction and Purification of Polysaccharides

**Figure 1:**
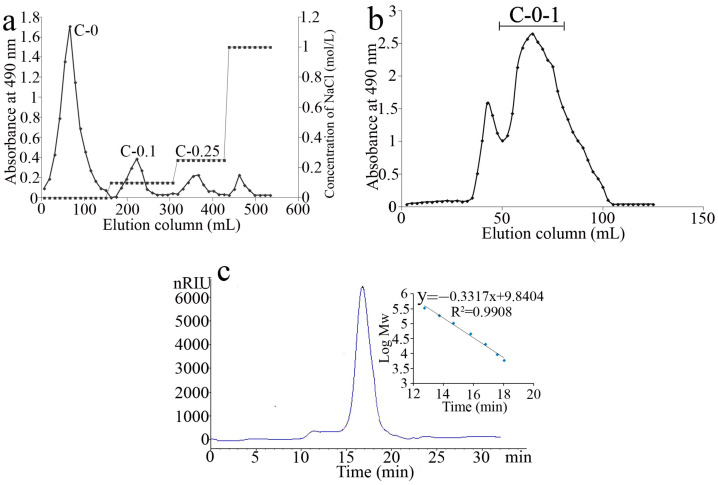
Elution profiles and molecular weight determination of crude polysaccharides. (**a**) Elution profile of the crude polysaccharide on anion-exchange column. (**b**) Purification of C-0 on GPC column. (**c**) HPGPC chromatogram of C-0-1 and standard curve for molecular weight.

The use of neutral proteinase-assisted extraction of *C. cicadae* mycelium to disrupt cell membranes and promote polysaccharide solubilization resulted in an improved yield of polysaccharides (7.3%), which was slightly more than that obtained from spores (5.6%) [17]. After elution with distilled water and different concentrations (0.1 M and 0.25 M) of NaCl in anion-exchange chromatography, three major peaks were obtained (Figure 1a), collected, and named as C-0, C-0.1, and C-0.25. C-0 (80% yield of the crude polysaccharide) was obtained and further purified on a Superdex 75 column to give the pure polysaccharide C-0-1 (75% of C-0) (Figure 1b).

### 2.2. Purity and Chemical Composition Analysis of C-0-1

HPGPC showed a single symmetric peak for C-0-1 in (Figure 1c), indicating uniform molecular weight distribution. The relative molecular weight of C-0-1 was calculated to be 23.3 kDa, which was similar to that of most polysaccharides from *C*. *sinensis*. Except for some glucan up to 1.0 ×10^6^ Da [18], the molecular weight of most of the polysaccharides from *C*. *sinensis* was in the range of approximately 10^3^ to 10^4^ Da [8]. A monosaccharide composition analysis showed that C-0-1 was a neutral heteropolysaccharide. It consisted mainly of mannose (Man), glucose (Glc), and galactose (Gal) in a molar ratio of 4:1:5. On summing up many different polysaccharides obtained from the fungus of *Cordyceps*, the monosaccharide compositions including Man, Gal, and/or Glc in various ratios were very common. Further, Glc and Man are the major monosaccharides in *C. sinensis* [7]. For C-0-1, its monosaccharide composition resembles the polysaccharides of *C. sinensis* with different resources. While Glc in C-0-1 had the minimum proportion, the abundance of Gal greatly increased.

### 2.3. Controlled Acid Hydrolysis and Methylation Analysis

The methylation analysis demonstrated that C-0-1 had diverse linkage patterns (Table 1). The ratio of the partial methylated alditol acetate derivatives was calculated by dividing each peak area from the total ion signals. The Gal residues were confirmed to be galactofuranosyl with non-reducing terminal Gal*f*, 1, 2-, and 1, 2,6-Gal*f* linkage styles; Man residues contained non-reducing terminal Man*p*, 1,2-, and 1, 2,6-Man*p* linkages; and Glc mainly had the 1, 4-Glc*p* linkage. Among these multiple linkage styles, the non-reducing terminals Gal*f*, 1, 2-Gal*f*, 1, 4-Glc*p*, 1, 2-Man*p*, and 1, 2,6-Man*p* were the main residues.

High contents of 1, 2,6-Gal*f* and 1, 2,6-Man*p* residues (approximately 40%) indicated that C-0-1 was a multi-branched polysaccharide. Because of its branched structure, controlled mild acid hydrolysis was performed to achieve the backbone information. After mild acid hydrolysis, the retained fraction C-0-1P was found to mainly contain Man, suggesting that C-0-1 had a core structure comprising of Man. Thus, the corresponding side chains contained Glc and Gal.

The methylation analysis result of the core structure C-0-1P showed that it primarily contained Man*p* residues including terminal, 1, 2-, 1, 2,6-, and minor 1, 6-Man*p* linkages. Compared with the proportion of 1, 2,6-Man*p* residue, that of 1, 2-Man*p* was found to be obviously higher, indicating that 1, 2-Man*p* was distributed in the main chain of the core structure.

The combination of these analyses contributed to further describe the structure outline as follows: (1) C-0-1 had a core structure with a 1, 2-Man*p* backbone and was branched at its 6-*O* positions by terminal Man*p* and 1, 6-Man*p* linkages, and (2) the branches linked to the core contained 1, 4-Glc*p*, the non-reducing terminal Gal*f*, and 1, 2-Gal*f* and 1, 2,6-Gal*f* linkages, which were located in the outer layer of the core structure.

### 2.4. NMR Analysis

**Figure 2:**
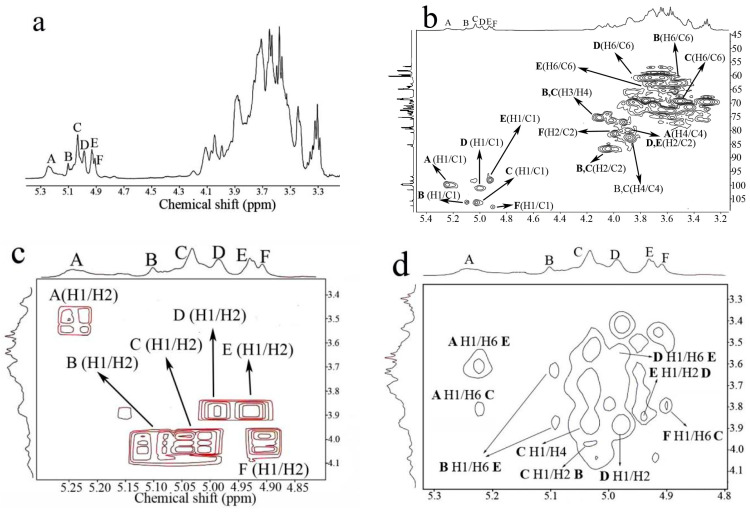
NMR spectra of C-0-1. (**a**) ^1^H NMR spectrum; (**b**) ^1^H–^13^C HSQC spectrum; (**c**) ^1^H–^1^H COSY spectrum in the anomeric region; and (**d**) NOESY spectrum.

The ^1^H NMR spectrum (Figure 2a) showed six major signals (designated as **A**–**F**) in the anomeric region. In correspondence, six residues were identified by COSY and HSQC spectrum. Their anomeric carbons and protons were also observed in HSQC (Figure 2b). The signals of **B** (5.1/106.7), **C** (5.03/106.9), and **F** (4.9/107.4) represented three different galactofuranose residues, which were determined by unusual low-field shifts of the anomeric carbon signals at about 107 ppm [19]. Moreover, the COSY spectrum (Figure 2c) revealed the relevance of H1/H2 of units **B** and **C** at 5.1/4.04 and 5.03/4.01, respectively. Next, HSQC confirmed their H2/C2 at 4.04/86 (unit **B**) and 4.01/86 (unit **C**). Similarly, the unusual shifts of C2 at 86 ppm indicated that both **B** and **C** had 2-*O* substitution. The downfield shift of C6 of **C** at 69 ppm, compared with that of C6 of **B** at 62.5 ppm also confirmed that **C** had 6-*O* substitution. These findings revealed that **B** was a (1→2)-β-D-Gal*f* residue and **C** was a (1→2, 6)-β-D-Gal*f* residue. Combined with a methylation analysis, **F** was deduced to be a terminal β-D-Gal*f* residue. Similarly, residues **D** and **E** were identified to be different types of mannose units. For residue **D,** the correlation signal of H1/C1 at 5.01/101.7 and H2/C2 at 3.88/80 confirmed the (1→2)-α-D-Man*p* unit. Based on the characteristic C2 (δ 80) and C6 (δ 67), unit **F** was deduced to be (1→2,6)-α-D-Man*p* [20]. The variation of C4 at δ 77.5 also confirmed that residue **A** was (1→4)-α-D-Glc*p*. Thus, the assignments of the six major residues were achieved based on the feature analysis and are presented in (Table 2).

The NOESY spectrum (Figure 2d) helped provide the correlation information between different units and helped achieve the major sugar sequences. The cross-peaks **D** H1/H2 **E** and **E** H1/H2 **D** revealed that (1→2)-α-D-Man*p* and (1→2,6)-α-D-Man*p* were linked together at the *O*-2 position. The signal **B** H1/H6 **E** suggested that (1→2)-β-D-Gal*f* was linked to the *O*-6 position of (1→2, 6)-α-D-Man*p*. **C** H1/H2 **B** and **F** H1/H6 **C** indicated that (1→2,6)-β-D-Gal*f* was linked to *O*-2 of (1→2)-β-D-Gal*f* and the non-reducing terminal β-D-Gal*f* was linked to (1→2,6)-β-D-Gal*f*. **A** H1/H6 **C** and **A** H1/H6 **C** indicated that the (1→4)-α-D-Glc*p* residue could be linked to *O*-6 of (1→2, 6)-β-D-Gal*f* or (1→2, 6)-α-D-Man*p*.

**Figure 3:**
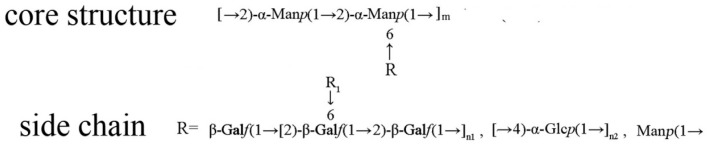
The possible structure of C-0-1.

The NMR analysis helped to draw the structure in detail based on the controlled acid hydrolysis and methylation analysis. C-0-1 was a comb-type and multi-branched galactoglucomannan with a mannan core. The mannan core had a backbone of 1, 2-α-Man*p* and approximately half of the backbone had branches at 6-*O* positions. The side chains of the backbone comprised terminal Man*p* and 1, 6-Man*p*. On the outer layer of the core structure, oligomers of (1→2)-β-D-Gal*f* and (1→4)-α-D-Glc*p* were linked to the 6-*O* positions of the backbone as a cover. In addition, 70% of the (1→2)-β-D-Gal*f* residues were substituted at the 6-*O* position by (1→4)-α-D-Glc*p* and terminal Gal*f*. Taking into consideration these analyses, a possible primary structure for C-0-1 was constructed (Figure 3). C-0-1 had a similar structure to that of the polysaccharide from the spores of *C. cicadae*, which has been reported before. The major difference was in the side chain. The side chain of the polysaccharide in the spores contained methylated (1→4)-α-D-Glc*p* and arabinose residues that were absent in C-0-1, and C-0-1 contained more (1→2, 6)-β-D-Gal*f*. This comparison indicated that polysaccharides in the spores and mycelium of the same fungus could have different structures in detail.

Heteropolysaccharides such as galactomannan and glucogalactomannan are typical polysaccharides in *C. sinensis.* These polysaccharides generally have a mannan core including Man*p* residues and Gal*f* side chains and represent a class of important bioactive polysaccharides in *C. sinensis* [8,21]. Although there are certain similarities, some differences in the details in the linkage mode are observed in these polysaccharides. A galactomannan in *C. sinensis* was reported to contain a (1→2)-D-Man*p* backbone and (1→5)-D-Gal*f* side chains [22]. A highly branched galactomannan had (1→2)-D-Man*p* residues, as well as (1→3), (1→5), and (1→6)-β-D-Gal*f* side chains [23]. In addition, a galactoglucomannan [24] from the mycelium of *C*. *sinensis* had a comb-like structure with (1→2)-D-Man*p* in the main chain and a non-reducing terminal α-D-Glc*p*, (1→5) and/or 6)-β-D-Gal*f* residues in the side chains. A galactomannan from a natural *C. sinensis* mainly consisted of a mannan backbone and Gal*f* side chains. The side chains were (1→5)-, (1→6)-, and the non-reducing terminal β-Gal*f* attached itself to the *O*-2 position of the main chain. The mannan skeleton was composed of (1→6)-α-D-Man*p* [18]. These examples revealed that most of these galactomannans have 1, 2-and/or 1, 6-α-Man*p* as the main chains and β-D-Gal*f* as the side chains. The major differences were the branching sites of the main chains and the linkages of β-D-Gal*f*. Overall, galactoglucomannan C-0-1 from *C. cicadae* was found to belong to the same kind of polysaccharides as *C. sinensis* (mannan core structure, Gal*f* side chain). Therefore, it is concluded that different polysaccharides from the genus *Cordyceps* have a similar structure (Table 3). Accordingly, *C. cicadae* can be a new alternative to *C. sinensis* to obtain bioactive polysaccharides.

### 2.5. Determination of Macrophage Activation Activity

**Figure 4:**
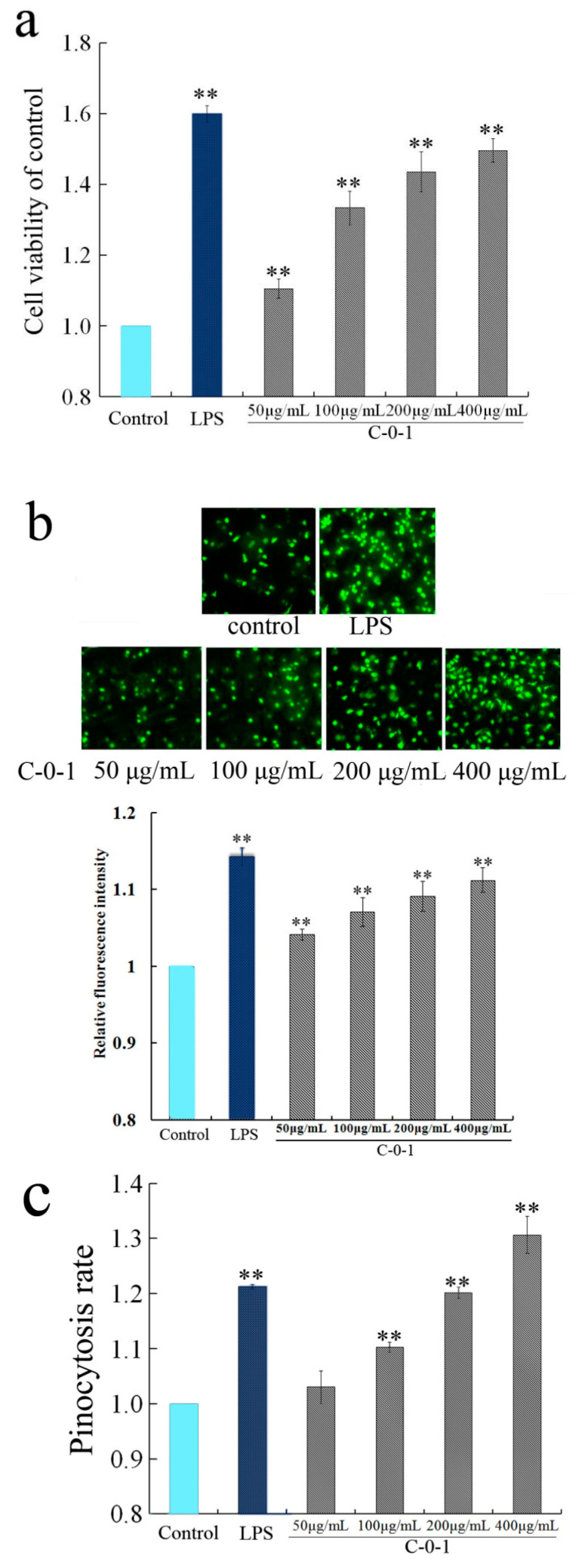

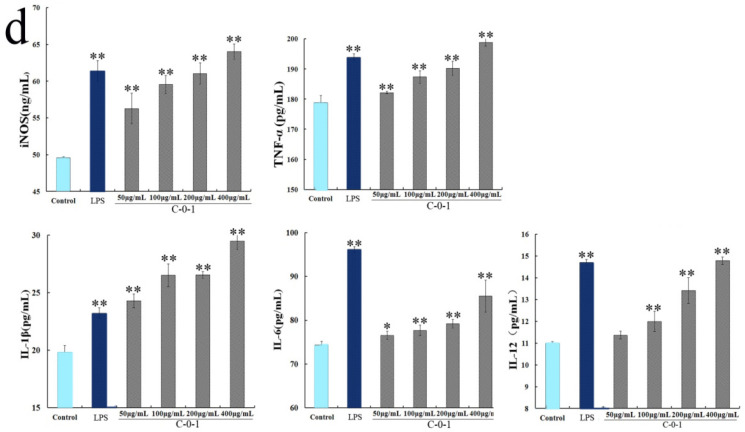
Effect of C-0-1on the activation of RAW264.7 cells. (**a**) Effect of C-0-1 on cell proliferation. (**b**) Effect of C-0-1 on ROS production. (**c**) Phagocytosed endocytic fluorescent-red latex beads stimulated by C-0-1. (**d**) Effect of C-0-1 on different concentrations of iNOS and major cytokines. The results were expressed as means ± SD (*n* = 3). * *p* < 0.05, ** *p* < 0.01 vs. control group.

The effect of polysaccharide C-0-1 from *C. cicadae* on the immunostimulatory activity and immune-related cytokines expression was investigated in RAW264.7 cells. C-0-1 exhibited significant proliferative activity at the concentrations of 50–400 µg/mL compared with the control LPS (Figure 4a). The neutral red uptake assay indicated that the pinocytic activity of RAW264.7 cells increased after treatment with C-0-1 (Figure 4c). These results indicated that C-0-1 strongly increased the pinocytic activity of macrophages.

Macrophages could be activated by increasing the levels of ROS, NO, iNOS, and several related cytokines when stimulated by pathological material or injury [25]. ROS production was measured using the fluorescent probe DCFH-DA. After treatment with LPS and C-0-1 at 50–400 µg/mL, the fluorescence intensity of DCF in treated cells was obviously stronger than the control group (Figure 4b), which indicated that cells treated with C-0-1 displayed up-regulated intracellular ROS production. At 400 µg/mL, C-0-1 could increase 10% of the fluorescence intensity. Many studies have indicated that ROS is involved in cell signaling and regulation responses, such as phagocytosis and apoptosis. ROS can serve as a common messenger that mediate the activation of the MAPKs/NF-κB signaling pathway during macrophage activation [26]. Given that C-0-1 could promote the production of ROS, it should be able to activate these signaling pathways in macrophages and increase the secretion of cytokines such as TNF-α. The iNOS enzyme in macrophages catalyzes NO production and release to regulates the inflammatory response [27]. The effects of C-0-1 at concentrations of 50–400 µg/mL on the iNOS secretion were observed. C-0-1 could increase iNOS production in a dose-dependent manner (Figure 4d). C-0-1 could also increase the production of critical cytokines including IL-β, IL-6, IL-12, and TNF-α, which participated in the macrophage activation [28] in a dose-dependent manner from a concentration of 50 to 400 µg/mL (Figure 4d) (*p* < 0.05).

### 2.6. Inhibition of Cytokine Production Using Anti-PRR Antibodies

The activation of macrophages is one of the first stages in the innate immune response to foreign microorganisms. Pathogen-induced recognition is mediated by a series of germline encoded pattern recognition receptors (PRRs), which can recognize conserved microbial components, known as pathogens, and associated molecular patterns (PAMPs) such as LPS, β-glucan, chitin, and mannoproteins.

The evidence suggests that polysaccharides from *C. cicadae* can activate macrophages, but no information is available on their related PRRs. Therefore, our study evaluated whether C-0-1 can interact with MR, TLR2, and TLR4 receptors on the surface of macrophages, and how it affects the cytokine production [29]. Macrophages were individually incubated with the monoclonal antibodies anti-MR, anti-TLR2, or anti-TLR4, which could preferentially bind to receptors and prevent the binding and identification of polysaccharide C-0-1.

**Figure 5:**
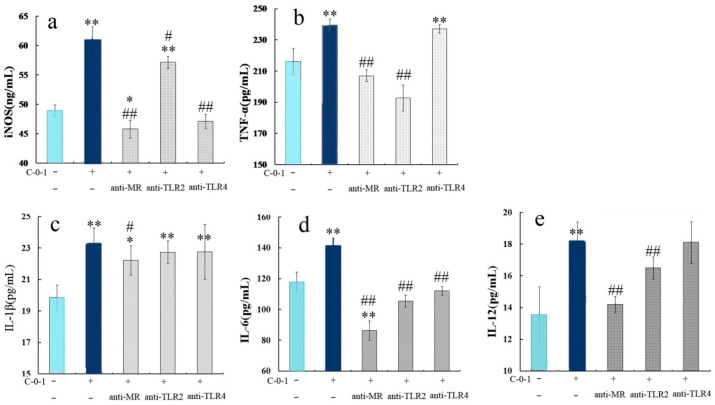
Binding and reaction of C-0-1 to RAW264.7 macrophages. RAW264.7 macrophages were incubated in the absence or presence of anti-MR, anti-TLR2, and anti-TLR4 MAbs individually. Subsequently, cells were treated with C-0-1, and the levels of cytokines were analyzed. (**a**) Variation in iNOS levels. (**b**–**e**) Variation of different cytokines levels ** *p* < 0.01 and * *p* < 0.05, compared to the negative control group; ## *p* < 0.01 and # *p* < 0.05, compared with the group treated with only C-0-1.

After pre-treatment with these three antibodies, the levels of iNOS and cytokines secretion in RAW 264.7 cells were found to decrease significantly, especially in the anti-MR group, indicating that all these three receptors, which participated in the reaction with C-0-1 and MR could be the most important receptor for C-0-1. It was also found that after pre-treatment with anti-TLR2, the level of TNF-α significantly varied in RAW 264.7 cells. In contrast, in the anti-TLR4 pretreatment group, the levels of cytokines were significantly reduced (Figure 5). Therefore, it was presumed that C-0-1 could recognize all the three receptors and participated in a series of immunomodulatory signaling pathways to increase the secretion of iNOS and different cytokines. MR receptors were involved in the regulation of iNOS, IL-6, IL-12, IL-1β, and TNF-α. Furthermore, the TLR2 receptor was mainly associated with the secretion of TNF-α, whereas TLR4 was involved in the iNOS secretion process. Polysaccharides from nature can directly activate the innate immune response as immunomodulators by mediating PRR signaling, e.g., the TLR groups. Many reports have demonstrated that mushroom polysaccharides activated macrophages with the help of TLR4. For instance, polysaccharide from *Paecilomyces cicadae* interacted with TLR4 and stimulated TNF-α and IL-1β production through the TLR4-related signaling pathway in macrophages [30]. Our study indicated that C-0-1 could activate macrophages with the help of multiple targets; this could be related to its complex structure. Based on data analysis, Glc, Gal, and Man are the three most common monosaccharides of the TLR-related polysaccharides [31]. In our study, mannan was the classic target ligand that bound to MR in the macrophage. These findings can explain the multiple targets of C-0-1. However, the effects on downstream pathways are more complex and deserve further study [32].

### 2.7. Effect of Polysaccharide on Immunomodulatory Activity in Mice

**Figure 6:**
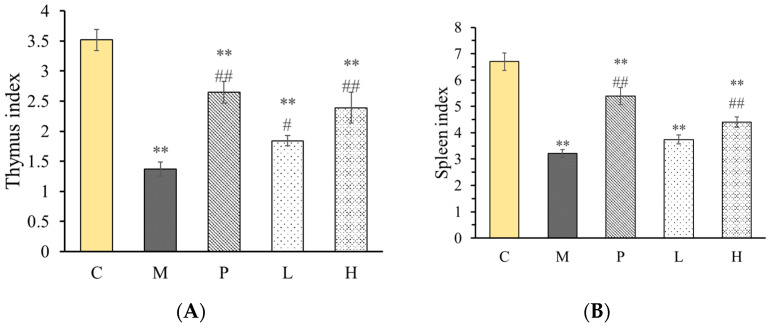
Influence of C-0-1 on immune organ index. (**A**) Thymus index and (**B**) spleen index data were presented as mean ± standard deviation (n = 6). ** p < 0.01, compared to the negative control group; *## p* < 0.01 and *# p* < 0.05, compared with the model group. C: control group; M: Cy-treated group; *p*: Cy + 40 mg/kg levamisole; L: Cy + 50 mg/kg C-0-1; and H: Cy + 200 mg/kg C-0-1.

Cyclophosphamide (Cy) is an important chemotherapeutic drug used for anti-tumor therapy but with serious side effects such as immunosuppression. In the investigation, the immunosuppressive mice induced by cyclophosphamide were used for investigating the immunomodulatory activity of C-0-1 in vivo. The condition of mammals' immune organs affects the level of immunity and the immune organ index, such as thymus and spleen indexes, which can be used to estimate the immune function. Levamisole, an immunomodulator that is used clinically with anti-cancer drugs to reduce the cytotoxic damage, was used as a positive drug. Additionally, the regulation effect of polysaccharide C-0-1 on immune organs of mice was studied by measuring the indexes of the thymus and spleen. According to Figure 6, the indexes of the thymus and spleen were significantly decreased in group M, the mice treated with Cy, illustrating that the immunity was disordered. However, the indexes of the spleen and thymus in group L and H were significantly higher than those in group M. The results showed that C-0-1, especially at a high dose, could effectively improve the immune organ index of mice, indicating that C-0-1 could regulate the atrophy of immune organs caused by Cy [33].

**Figure 7:**
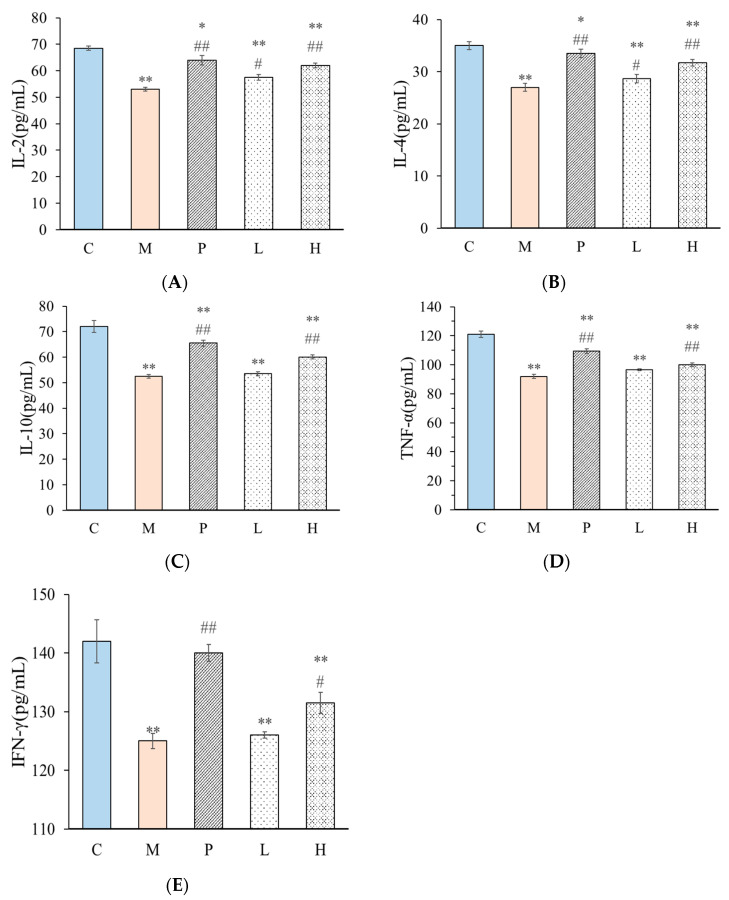
Effects of C-0-1 on IL-2 (**A**), IL-4 (**B**), IL-10 (**C**), TNF-α (**D**) and IFN-γ (**E**) contents of ileal inflammatory factors in immunosuppressed mice. Data were presented as mean ± standard deviation (*n* = 6). ** p < 0.01 and * p < 0.05, compared to the negative control group; *## p* < 0.01 and *# p* < 0.05, compared with the model group. C: control group; M: Cy-treated group; *p*: Cy + 40 mg/kg levamisole; L: Cy + 50 mg/kg C-0-1; and H: Cy + 200 mg/kg C-0-1.

The secretion of related cytokines such as IL-2, IL-4, IL-10, TNF-α, and IFN-γ plays an important role in the improvement of immune function. The secretion levels of serum inflammatory factors in the serum of mice were detected by ELISA kit, as shown in Figure 7. Compared with group C, the secretion levels of IL-2, IL-4, IL-10, TNF-α, and IFN-γ in group M significantly decreased. The intake of C-0-1 significantly increased the secretion of related cytokines in immunosuppressed mice, and the effect increased in a dose-dependent manner.

T helper 1 (Th1) and T helper 2 (Th2) cells are characterized by specific cytokine signatures. Th1 mainly secretes IL-2, IL-12, TNF-α, and IFN-γ, while Th2 mainly secretes IL-4, IL-5, IL-10, and IL-13. IL-2 and IFN-γ are considered to be hallmark Th1 cytokines while IL-4 is the hallmark Th2 cytokine. These factors affect each other and work together to maintain the dynamic balance of the body’s immune system. The Th1/Th2 balance is the major framework used to address adaptive immunity and describe the state of immune balance [34]. The Th1/Th2 balance in mice was calculated according to the formula, and the results are shown in Table 4. The serum Th1/Th2 in mice in the model group M significantly increased the balance value of cytokines compared with the blank group C. It is proven that Cy can break the balance value of cytokine secretion in normal mice, thus causing immune dysfunction. The positive drug group P can significantly change the serum Th1/Th2 value of mice after modeling and restore the immune balance in mice. When C-0-1 was administered at low and high doses, the concentrations of Th1/Th2 in serum of mice were 3.36 ± 0.01 and 3.17 ± 0.10, respectively, and they showed a concentration-dependent decrease. Thus, it was believed that polysaccharide C-0-1 can improve the secretion of cytokines in immunosuppressive mice, so as to regulate the immune system and gradually restore the balance of the damaged immune environment.

The polysaccharides from *C. sinensis* have abundant biological activity, and an especially remarkable immunomodulatory activity. Many studies have shown that these polysaccharides can stimulate the phagocytic function of macrophages, initiate cellular and humoral immune responses, and enhance the spleen index and thymus index. They can also significantly enhance the neutral red uptake of peritoneal macrophages and stimulate the release of related cytokines, such as inducing the production of TNF-α, IL-6, and IL-10 dose-dependently, demonstrating their immunomodulatory role [7]. Our result indicated that C-0-1 from *C. cicadae* had a similar structure and immunomodulatory activity to the polysaccharides from *C. sinensis*.

## 3. Materials and Methods

### 3.1. Materials

The mycelium of natural *C. cicadae* was obtained from the bamboo cicada *(Platylomia pieli* Kato) in the bamboo forest of Zhoushan archipelago, East China, in July. RAW264.7 cells were purchased from the Type Culture Collection of Chinese Academy of Sciences (Shanghai, China).

### 3.2. Extraction and Purification of Polysaccharides

Dried and defatted mycelium powder of *C. cicadae* was extracted by enzyme-assisted method to obtain the polysaccharide. In brief, the defatted mycelium (5%, *w*/*v*) and neutral proteinase (0.8%, *w*/*v*) were added to distilled water at 45 °C and incubated for 2 h. After enzymolysis, the solution was boiled for 30 min, and the supernatant was collected by centrifugation and concentrated by rotary evaporation to 1/4 of the original volume. Subsequently, four times the volume of ethanol was added to the solution and incubated overnight at 4 °C. The precipitate was collected by centrifugation (1760× *g*, 15 min), dissolved, dialyzed, and lyophilized to achieve the crude polysaccharide product [35].

The crude polysaccharide was first purified using the Q Sepharose Fast Flow anion-exchange column (300 × 30 mm) and eluted successively with 0, 0.1, 0.25, and 1 M NaCl for 2 CV of each gradient. The C-0 fraction, which was eluted with water and belonged to the most abundant sugar-containing fraction, was obtained, and lyophilized. It was further purified using a gel permeation column (Superdex 75, 70 × 2 cm, GE Healthcare, Chicago, IL, USA) eluted with 0.2 M NH_4_HCO_3_ at 0.3 mL/min. A major peak was then observed and named C-0-1.

### 3.3. Purity, and Chemical Composition Analysis

Purity and molecular weight distribution were determined by high-performance gel permeation chromatography (HPGPC) method. The monosaccharide compositions were determined by 1-phenyl-3-methyl-5-pyrazolone (PMP) pre-column derivatization HPLC method after complete acid hydrolysis [36].

### 3.4. Controlled Acid Hydrolysis and Methylation Analysis

According to a previously reported method with some modifications [37,38], C-0-1 (10 mg) was hydrolyzed with 0.2 M TFA (105 °C, 2 h). The hydrolysate was evaporated to dryness and re-dissolved in 75% ethanol. The soluble and insoluble products were separated by centrifugation (3600× *g*, 10 min), vacuum-dried, and labeled as C-0-1S and C-0-1P, respectively, followed by monosaccharide compositional analysis of C-0-1P as the method described above [39].

In addition to monosaccharide composition analysis, the glycosidic linkage styles of C-0-1 and the hydrolysate (C-0-1P) were further investigated using methylation and GC-MS analysis. The methylation of C-0-1 and C-0-1P was performed according to a modified Hakomori method [1].

### 3.5. NMR Spectroscopy

After active protons such as -OH were exchanged, freeze-dried C-0-1 (60 mg) was dissolved in 400 μL of D2O and transferred to a 5 mm NMR tube. NMR was performed on a Bruker 800-MHz NMR spectrometer at a temperature of 298 K.

### 3.6. Determination of Macrophage Activation

#### 3.6.1. Effects of C-0-1 on RAW 264.7 Cells Viability

To study the toxicity of C-0-1on RAW 264.7 cells, their viability was assessed via MTT assay. The toxicity of different concentrations of C-0-1 (50–400 µg/mL) on RAW 264.7 cells was studied and analyzed using lipopolysaccharide (LPS) (2 µg/mL) for comparison [40].

#### 3.6.2. Determination of ROS Levels in RAW 264.7 Cells

The effect of LPS (2 µg/mL) and C-0-1 (50–400 µg/mL) on ROS production in RAW264.7 cells was measured using the fluorescent probe 2,7-dichlorodihydrofluorescein dilacerate (DCFH-DA) [41].

#### 3.6.3. Determination of Phagocytic Uptake

The effects of LPS (2 µg/mL) and polysaccharide C-0-1 (50–400 µg/mL) on the phagocytic uptake activity of macrophages was determined using fluorescent-red latex beads (2 µm) and neutral red [30].

#### 3.6.4. Measurement of Inducible Nitric Oxide Synthase (iNOS) Production

RAW264.7 cells were pre-incubated at density of 1 × 106 cells/mL for 24 h. Next, LPS (2 µg/mL) and C-0-1 (50–400 µg/mL) were added and incubated at 37 °C for 24 h. Cell culture supernatants were collected and stored at −20 °C. The iNOS level was determined by ELISA kit (Nanjing Jiancheng Technology, Nanjing, China) [41].

#### 3.6.5. Cytokine Assays

The RAW264.7 cells were pre-incubated at density of 5 × 105 cells/mL for 24 h with LPS (2 µg/mL) and C-0-1 as mentioned above. The production of IL-1β, IL-6, IL-12, and TNF-α in the cell culture supernatants was analyzed using an ELISA kit [42].

#### 3.6.6. Cytokine Production Inhibition Using Anti-PRR Antibodies

After the RAW264.7 cells were pre-incubated in a 96-well plate and the culture media was removed, 100 µL anti-MR anti-TLR2 and anti-TLR4 MAbs (5 µg/mL) were added to each well 1 h before adding C-0-1 polysaccharides (200 µL, 200 µg/mL) and incubated for 24 h at 37 °C. After incubation, the cytokine productions were analyzed as described above [43,44].

#### 3.6.7. Statistical Analysis

All data were processed statistically, and divergences are presented as mean ± SD. SPSS 16.0 for Windows (Pearson Co., Beijing, China) was used to compare the differences between various treatment groups. Tukey’s post hoc test was used for variance analysis and *p* < 0.05 indicated significance differences.

### 3.7. Immunomodulatory Activity In Vivo

SPF-free ICR female mice aged 6–8 weeks with the body weight of 20 ± 0.2 g were chosen to screen the immunomodulatory activity in vivo. All animal testing procedures were approved by the Animal Ethics Committee of Zhejiang Ocean University.

After placed in a light–dark cycle at room temperature for 12 h and 1 week of adaptive breeding, the mice were randomly divided into 5 groups (n = 6): control group (C), model group (M), positive control group (P), low dose group (L), and high dose group (H). From day 1 to day 3, the immunosuppressed was established. Mice were given administration as follows: control group (C): intragastric normal saline; the other groups: intraperitoneal injection with cyclophosphamide (80 mg/kg BW/d). From day 4 to day 10, control group (C) and model group (M): normal saline; positive control group (P): levamisole (40 mg/kg BW/d); low dose group (L): C-0-1(50 mg/kg BW/d); and high dose group (H): C-0-1 (200 mg/kg BW/d).

The body weight of mice in each group was recorded before death, and the eye blood was collected quickly before the neck was cut off. Then, the thymus and spleen of the mice were collected, washed with PBS buffer solution for 3 times, dried with filter paper, weighed, labeled, and stored in a refrigerator at −80 °C. The spleen index and thymus index were evaluated by the following formula: spleen (thymus) index = the weights of spleen (thymus) (mg)/the body weight (g) × 10.

To study the effects of the polysaccharide C-0-1 on immunomodulatory activity in mice, the blood taken from the eyeballs of mice was centrifuged at 3000 r/min for 10 min immediately after blood collection, and the upper serum was taken. The contents of IL-2, IL-4, IL-10, IFN-γ, and TNF-α in serum of mice were detected according to the Elisa kit instructions, and Th1/Th2 was calculated according to the formula: Th1/Th2 = IFN-γ + TNF-α + IL-2/IL-4 + IL-10 [45].

## 4. Conclusions

A galactoglucomannan C-0-1 (23.3 kDa) was obtained from the mycelium of *C. cicadae* C-0-1, which exhibits good immunomodulatory activity on macrophages by enhancing the iNOS production and the secretion of the major inflammatory cytokines in RAW 264.7 cells. The main PRR that participated in the recognition between C-0-1 and macrophages is the MR receptor. TLR4 and TLR2 also participated in part of the recognition. C-0-1 also regulated the secretion of serum-related cytokines (IFN-γ, TNF-α, IL-2, IL-4, and IL-10) and affected the balance of Th1/Th2 in the immunosuppressed mice. All the results demonstrated that C-0-1 had immunomodulatory activity and can regulate immunosuppression.

Sufficient evidence indicates that *C. cicadae* has medicinal benefits and contains bioactive compounds similar to those found in *C. sinensis*. Our research further shows that the major polysaccharides from *C. cicadae* are largely the same as those from *C. sinensis* in terms of structure and immunomodulatory activity. A far-ranging distribution, mild growing conditions, and mature cultivation methods for *C. cicadae* ensured the demands and sustainability of its uses. Thus, in terms of polysaccharides, *C. cicadae* is an ideal alternative source to *C. sinensis*.

## Figures and Tables

**Table 1:** GC–MS data of partial methylated alditol acetate derivative of C-0-1 and its core structure C-0-1P.

Methylation Product	Linkage Type	Main MS (*m*/*z*)	Molar Ratio (%)	
			C-0-1	C-0-1P
1,4-Ac_2_-2,3,5,6-Me_4_-D-Gal	Gal*f*(1→	117,161,205,277	13.4	—
1,5-Ac_2_-2,3,4,6-Me_4_-D-Man	Man*p*(1→	117,129,145,161,205	5.2	—
1,2,5-Ac_3_-3,4,6-Me_3_-D-Man	→2)Man*p*(1→	129,161,189	14.8	74.5
1,4,5-Ac_3_-2,3,6-Me_3_-D-Glc	→4)Glc*p*-(1→	113,117,131,161,173,233	11.2	8.9
1,2,4-Ac_3_-3,5,6-Me_3_-D-Gal	→2)Gal*f*(1→	117,129,143,161	8.8	3.9
1,5,6-Ac_3_-2,3,4-Me_3_-D-Man	→6)Man*p*(1→	117,129,161,189	4.6	—
1,2,5,6-Ac_4_-3,4-Me_2_-D-Man	→2,6)Man*p*(1→	129,189	18.9	12.7
1,2,4,6-Ac_4_-3,5-Me_3_-D-Gal	→2,6)Gal*f*(1→	117,129,189	23.2	—

**Table 2:** ^1^H and ^13^C NMR chemical shifts (δ) for the residues of galactoglucomannan C-0-1.

Residue	H1/C1	H2/C2	H3/C3	H4/C4	H5/C5	H6/C6
**A** (1→4)-α-D-Glc*p*	5.25	3.53	3.7	3.58	—	3.78, 3.63
	99.0	71.7	74.0	77.5	—	60.6
**B** (1→2)-β-D-Gal*f*	5.1	4.04	4.1	3.89	3.75	3.55, 3.76
	106.7	86	75.1	82.4	70	62.5
**C** (1→2,6)-β-D-Gal*f*	5.03	4.01	4.1	3.89	3.6	3.56, 3.8
	106.9	86	75.1	82.4	70.8	69
**D** (1→2)-α-D-Man*p*	5.01	3.88	3.76	3.35	3.6	3.65, 3.75
	101.7	80	70.6	69	74	61
**E** (1→2,6)-α-D-Man*p*	4.92	3.88	3.76	3.35	3.6	3.9, 3.6
	98.0	80	70.6	69	74	67
**F** β-D-Gal*f*(1→	4.9	4.01	4.07	3.94	3.75	3.63, —
	107.4	82	78.5	82.7	70.9	62.3

**Table 3:** Chemical structures of galactomannan originated from *Cordyceps sinensis* fungi.

Fungus	Polysaccharide Resource	Component	Molecular Weight	Linkages	Reference
*Cordyceps sinensis*	Ascocarps	Man:Gal = 3:5	23 kDa	Backbone: (1→2) and (1→6) -α-D-Manp. Side chain: non-reducing terminal β-Gal*f*, (1→5)-β-Gal*f*, and non-reducing terminal α-D-Man*p*. Branch point: *O*-6/*O*-4.	[21]
*Cordyceps sinensis*	Nature Ascocarps	Man:Glc:Gal = 24:7:69	7.2 kDa	Backbone: (1→6)-α-D-Man*p*. Side chain: non-reducing terminal, (1→5), (1→6)-β-D-Gal*f*, (1→2)-α-D-Man*p*, and non-reducing terminal α-D-Man*p*. Branch point: *O*-2/*O*-4.	[18]
*Cordyceps sinensis*	Ascocarps	Man:Gal = 1:1	-	Backbone: (1→2)-α-D-Man*p*. Side chain: non-reducing terminal, (1→3), (1→5), (1→6)-β-Gal*f*, and non-reducing terminal α-D-Man*p*. Branch point: *O*-6/*O*-4.	[23]
*Cordyceps sinensis*	Cultured mycelium	Man:Glc:Gal = 24:33:43	15 kDa	Backbone: (1→2)-α-D-Man*p*. Side chain: terminal, (1→5), (1→6)-β-Gal*f*, and non-reducing terminal α-D-Glc*p.*	[24]

**Table 4:** Effects of C-0-1 on Th1/Th2.

	NC	MC	PC	C-L	C-H
Th1/Th2	3.13 ± 0.11	3.42 ± 0.01 **	3.18 ± 0.14 ##	3.36 ± 0.01 **	3.17 ± 0.10 ##

** p < 0.01, compared to the negative control group; *## p* < 0.01, compared with the model group. C: control group; M: Cy-treated group; P: Cy + 40 mg/kg levamisole; L: Cy + 50 mg/kg C-0-1; and H: Cy + 200 mg/kg C-0-1.

## Data Availability

The data presented in this study are available in the article.

## Sample Availability

Not available.
